# Intellectual property meets transdisciplinary co-design: prioritizing responsiveness in the production of new AgTech through located response-ability

**DOI:** 10.1007/s10460-022-10378-3

**Published:** 2022-11-01

**Authors:** Karly Ann Burch, Dawn Nafus, Katharine Legun, Laurens Klerkx

**Affiliations:** 1 University of Otago, P.O. Box 56, Dunedin, 9054 New Zealand; 2 Intel, Portland, USA; 3 Wageningen University, P.O. Box 9101, 6700 HB Wageningen, The Netherlands

**Keywords:** Intellectual property, Collaborative design (co-design), Artificial intelligence (AI) and robotics, Digital agriculture, Agriculture 4.0, Located accountability and located response-ability, Responsiveness, Feminist STS, Responsible research and innovation

## Abstract

This paper explores the complex relationship between intellectual property (IP) and the transdisciplinary collaborative design (co-design) of new digital technologies for agriculture (AgTech). More specifically, it explores how prioritizing the capturing of IP as a central researcher responsibility can cause disruptions to research relationships and project outcomes. We argue that boundary-making processes associated with IP create a particular context through which responsibility can, and must, be located and cultivated by researchers working within transdisciplinary collaborations. We draw from interview data and situated IP practices from a transdisciplinary co-design project in Aotearoa New Zealand to illustrate how IP is a fluid *boundary-requiring-and-producing object* that impels researchers into its management, and produces tensions that need to be noticed and skillfully navigated within research relations. We propose *located response-ability* as a conceptual tool and practice to reposition IP within the relations that make up a transdisciplinary co-design project, as opposed to prioritizing IP by default without recognizing its possible impacts on collaborative relations and other project aims and accountabilities. This can support researchers practicing responsible innovation in making everyday decisions on how to protect potential IP without disrupting the collaborative relations that make the creation of potential IP possible, and the existence of protected IP relevant and beneficial to project collaborators and wider societal actors. This may help to ensure that societal benefits can be generated, and positive science–society relationships prioritized and preserved, in the design of new AgTech.

## Introduction

My first week on the job, I [Karly] found myself on a vineyard on the North Island of Aotearoa New Zealand.^1^ It was the middle of winter and the vines, now filled with buds, were ready to be pruned. Engineers and computer scientists were interacting with vines, farm managers and agricultural consultants, collaboratively imagining how the new digital agricultural technologies (AgTech) the project was developing could support growers and agricultural workers in their everyday work. As an embedded social scientist, I was an AgTech novice and spent my time in the field asking my new colleagues questions about the technologies the project was developing: What were they? How did they work? What could they do? How does a robot know what a vine is anyways? I was excited to share details about the technologies with the growers and agricultural workers I would interview, to help them to imagine the possible roles these particular technologies might play in their future work. As I was planning my fieldwork, I attended my first project meeting. An Intellectual Property (IP) registry was mentioned at the meeting, and the meeting itself was followed by a presentation on IP led by representatives from the lead university’s research office. The presentation described the forms of IP that were considered most valuable (patents) and my contractual obligation to prevent potential IP from being shared publicly before it was protected (this included anything shared in my interviews). Turning back to my research plans, I now had a new participant to consider in my fieldwork: potential IP. Was there a chance that I would accidentally discuss potential IP in my interviews? What would I need to censor and what could I share? Beyond my own fieldwork, how does transdisciplinary collaborative design (co-design) take place when researchers are unable to share technological details and prototypes with research collaborators and other possible end-users? If what growers or agricultural workers had to say could substantially inform how the IP should work, is showing it to them after patenting too late? How do other project colleagues navigate these tensions in their everyday research?

This paper explores the complex relationship between IP and the transdisciplinary co-design of new AgTech. More specifically, it explores ways that prioritizing the capturing of IP as a central researcher responsibility can cause subtle and not-so-subtle disruptions to collaborative research relationships and project outcomes, including the integrity and value of technologies and their industry and societal relevance. Our interest in IP comes from critically observing low-stakes IP-induced tensions emerging within the MaaraTech Project—a large-scale New Zealand Ministry of Business, Innovation and Employment (MBIE)-funded transdisciplinary project co-designing robotic and human-assist technologies with artificial intelligence (AI) capabilities for use in high-value fruit industries in Aotearoa New Zealand.

Much of the literature discussing IP in agriculture focuses on the social and environmental consequences that emerge when protected IP enters and reorganizes agrifood worlds, as opposed to the role of IP in shaping AgTech design processes. These literatures highlight how IP participates in concentrating power in the hands of agrifood, AgTech and BigTech firms, disempowering farmers and taking away their agency in regards to their equipment (e.g., preventing tinkering and self-repair), and generally contributing to widening power asymmetries between technology and data owners (e.g., BigTech, AgTech and agribusiness firms) and technology users and data producers (e.g., farmers, agricultural workers, etc.) (Bronson 2019; Bronson and Knezevic 2019; Bronson and Sengers 2022; Bronson et al. 2021; Carbonell 2016; Carolan 2017, 2018, 2020; Fraser 2019, 2020; Higgins et al. 2017; Rotz et al. 2019b; Stock and Gardezi 2021).

The need for technology owners to protect IP has led to a situation in which farmers and other possible end-users (e.g., agricultural workers) often have little to no input into technology development processes which tend to be directed by industry interests—a “top-down” as opposed to “bottom-up” approach, which often results in the creation of technologies that do not actually support farmers or solve the problems they face in ways that are accurate or effective (Rotz et al. 2019a, b, p. 212). As a result, technology development processes that center profit often result in the development of technologies that farmers are not interested in adopting (Lindblom et al. 2017). Put differently, while IP might make investment in research and development of new AgTech attractive (Carolan 2010; Blakeney 2020), it can also maintain clear boundaries between technology developers and possible users (and the sites of technology use), leading to the production of technologies that are not societally relevant or fit for purpose—and thus technologies that farmers do not want to adopt. This leads to what some refer to as the “problem of implementation,” or a lack of farmer adoption of new AgTech (Lindblom et al. 2017, p. 312).

Transdisciplinary co-design has become one of many methods aimed at disrupting asymmetrical power dynamics between developers and users. With its roots in early participatory design movements emerging out of Europe in the 1970s (e.g., Simonsen and Robertson 2012), transdisciplinary co-design aims to create socially beneficial and relevant technologies through including possible end-users in technology design processes (see, for example, Berthet et al. 2018; Botha et al. 2014; Bos et al. 2009; Cerf et al. 2012). Recently, scholars have been advocating for the use of transdisciplinary co-design in the field of AgTech (Kenny and Regan 2021; Lioutas and Charatsari 2021; McCampbell et al. 2021b), and such considerations were central in the development of the MaaraTech Project. Within MaaraTech, project leadership’s commitment to transdisciplinary co-design materialized as a team of interdisciplinary scholars and agricultural consultants engaging with possible end-users: industry partners and growers in meetings and co-design workshops, and (eventually) agricultural supervisors, trainers and workers through interviews with social scientists (see Burch and Legun 2021; Burch et al. 2022).

Some scholars have discussed the general tensions IP can cause in co-design projects involving university researchers and industry representatives, where IP agreements can be created to allow for open deliberation about potential IP among project collaborators (e.g., Okamuro and Nishimura 2013). These include tensions that arise from academic research becoming censored, inventions becoming “locked up” (i.e., development foreclosed due to tensions within co-ownership models) (Kneller et al. 2014, p. 13), or differences in “IP ownership aggressiveness” affecting the design and utility of IP agreements (Gretsch et al. 2020). While these insights are important, there is little written about the impacts of IP within projects that engage with people who may *not* be granted the right to know about potential IP based on a project’s particular IP agreement. Thus, these previously described tensions do not help us to understand *how* dynamics around IP unfold in everyday research practices and social interactions within transdisciplinary co-design projects. This leads to a number of questions about how IP might affect researchers’ abilities to be responsible collaborators as they engage in the everyday work of transdisciplinary co-design. This is because being a responsible collaborator in transdisciplinary co-design requires researchers engage in the difficult work of achieving multiple project aims and accountabilities (e.g., producing and protecting potential IP) while they remain responsive to research collaborators and wider societal actors (e.g., remaining responsive to people with whom they can and cannot discuss potential IP).

Responsibility has been denoted as a key issue in AgTech innovation such as precision technologies, AI and robotics (e.g., Rose and Chilvers 2018; Bronson 2019; Eastwood et al. 2019; Newton et al. 2020; Rose et al. 2021; van der Burg et al. 2019). Our research extends this line of thinking by exploring how IP can, and must, be practically and responsibly negotiated within situated transdisciplinary co-design processes. Such an approach allows for recognizing the challenges associated with IP management, everyday strategies that make sense within a particular project, and the potential societal implications of the outcomes of those strategies. This focus aligns our work with other research pointing to the gaps between theory and practice, and the practical, everyday barriers experienced by researchers in real time, as they try to make technology development more responsible and participatory (see Borch and Throne-Holst 2021; Kuzma and Roberts 2018; Kyng 2010; Liboiron 2017; Molla et al. 2018; Nathan 2015; Timmermans 2017; Ribeiro et al. 2018; Stahl et al. 2013; and Stilgoe et al. 2013 for technology development in general; see Regan 2021; Fielke et al. 2021; Lioutas and Charatsari 2021; McCampbell et al. 2021b for the case of digital agriculture). It also puts our research in dialogue with scholars who advocate for discussing responsible innovation in the context of everyday research taking place within “academic capitalism” (Glerup et al. 2017; Hackett 2014), and how researchers can respond when aims of economic productivity and societal benefit—both promoted through the responsible research and innovation framework—conflict in everyday research practice (de Saille 2015).

Therefore, in this paper we empirically examine team members’ IP-related reflections, as well as situated IP processes and outcomes emerging within the MaaraTech Project. We undertake this examination to identify challenges and shortcomings associated with prioritizing the capturing of IP as a central researcher responsibility, focusing on the possible technical and social implications of this (often default) prioritization. We use these findings to advocate for the use of *located response-ability* to support researchers engaged in transdisciplinary co-design to more skillfully notice and navigate IP-induced tensions within their situated design processes. While our findings can support those engaged in transdisciplinary co-design in general, they have particular implications for the future development of AgTech which are being designed within and for dynamic farming systems—each with their particular landscapes, institutional entanglements, labor relations, accountabilities and responsibilities (Legun and Burch 2021; see also Eastwood et al. 2019; Legun et al. this special issue; Prause 2021; Regan 2021; Rijswijk et al. 2021).

In our work, we show how IP is a fluid *boundary-requiring-and-producing object*: potential IP requires the creation of specific boundaries in order to become protected IP; protected IP creates boundaries between owners and users which are legally enforceable through intellectual property rights. We refer to IP’s *boundary-requiring-and-producing* nature as fluid because these boundaries are not fixed, but constantly emerging and transforming, particularly before, but also after, potential IP is protected (Carolan 2010). We understand and examine these boundaries through the lens of located response-ability*,* which combines insights from Suchman’s (2002) conceptualization of “located accountability”—described further in section three—with Barad’s (2007) conceptualizations of response-ability from the field of feminist science and technology studies (STS). Located response-ability also aligns with discussions about navigating “compromised agency” in technology design emerging from the field of feminist and anti-colonial STS (Liboiron 2017).

Dominant conceptualizations of responsibility often distinguish between normative or moral responsibilities to minimize negative outcomes before they happen (ex-ante responsibility), and a duty to respond when something goes wrong (ex-post responsibility) (Rijswijk et al. 2021). Through these conceptualizations, responsibility is considered to be a static social designation that is imagined, enacted or “taken” in discrete moments. As a result, the active, relational awareness of who is responding to who/what (and who/what might be preventing that response) becomes an afterthought, made known only as the responsible party is located and made answerable for something. Response-ability flips this process on its head: beginning with the premise that people and other more-than-human actors (e.g., potential IP, IP agreements, plants, robots) are always in relation and are, thus, always responding (or being prohibited from responding) within complex relational entanglements. This inability to be outside of relations means that questions of politics and ethics are present in everyday encounters, emerging through everyday actions such as who/what people choose to respond to, or are capable of responding to, at any given moment. This creates a situation where each person becomes responsible for “the exclusions that [they] participate in enacting” (Barad 2007, p. 394), instead of responsibility being seen as something decided at a later time, or something only social scientists need to grapple with within transdisciplinary projects.

While concepts such as “responsibilisation” are being deployed to support people engaged in complex “socio-cyber-physical systems” to notice their responsibilities for various social and material phenomena (e.g., Rijswijk et al. 2021), response-ability focuses on the situated relations within which particular responsibilities (and corresponding abilities to respond) emerge and are negotiated in practice (see also Lioutas and Charatsari 2021). We believe this focus on the everyday, situated relations of transdisciplinary co-design can support researchers to notice how their abilities to respond (response-abilities) within everyday encounters shapes their abilities to be responsive within research relationships and to wider societal actors. This, in turn, shapes their abilities to be responsible and accountable for their actions and the outcomes of technology design processes (and to change how they respond if the outcomes are not in alignment with their aims, accountabilities or values). While heightened awareness about available response-abilities does not guarantee intended results, it can provide opportunities for researchers to be strategic, especially when their agencies are compromised (Liboiron 2017). Thus, response-ability turns the focus away from responsibility as an imagined state or outcome, to responsibility as an active, everyday practice where researcher response-abilities may shift depending on the particular place they are located, situation they are in and particular actors (human and more-than-human) involved.

As a theoretical anchor of this paper, response-ability is a conceptual tool and practice that invites researchers engaged in transdisciplinary co-design to recognize the everyday, relational aspects of technology development and transdisciplinary collaboration, and to consciously participate in shaping the social and material relations that are brought together to achieve the specific aims and uphold the specific accountabilities of a project. In the MaaraTech Project, this included relations among researchers, industry partners, growers, potential IP, apple trees, blueberry bushes, winegrape vines, robot arms, computer vision cameras, IP agreements, ethics consent forms, among myriad other human and more-than-human entities. Response-ability is a playful, yet serious, term that invites researchers to notice two aspects: (1) how they relate (to whom/what they are obliged to respond to within a set of relations); and (2) the boundaries that may shape or limit their abilities to respond. Barad (2007, p. 148) uses the term “agential cuts” to describe how such boundaries are not given, but made through situated practices (e.g., when a scientist transforms a subject into an object of scientific study, she creates a new boundary between them). In this paper, we use the concepts of response-ability and boundaries to notice and articulate *how* IP-induced tensions emerge within technology design processes, and to find ways to address and anticipate these tensions to ensure they do not impose detrimental costs to the relations that make societally relevant and beneficial outputs possible within a transdisciplinary co-design project.

Our analysis highlights how the boundaries required to protect potential IP can restrict responsiveness to both industry and non-industry collaborators within transdisciplinary co-design processes. This is because the boundaries required to protect potential IP shape forms of interaction and negotiation within the relations of technology production. This can emerge as boundaries between, for example, Western and non-Western conceptualizations of ownership and property; technology developers and possible end-users; sites of production and sites of use; owners and customers; among others. We argue that boundary-making processes associated with IP create a particular context through which responsibility can, and must, be located and cultivated in everyday research practices, particularly in the context of academic capitalism where IP is a regular actor within everyday research encounters (Glerup et al. 2017). We propose using located response-ability as a conceptual tool and practice to reposition IP within the relations that make up a transdisciplinary co-design project—as opposed to prioritizing IP without recognizing its possible impacts on collaborative relations, other project aims and accountabilities, the societal benefits of technologies and wider science–society relationships. This can support researchers engaged in these projects to make conscious decisions about IP in their everyday work. Particularly, about how to protect potential IP while also protecting the collaborative relations that make the creation of potential IP possible, and the existence of protected IP relevant and beneficial to project collaborators and wider societal actors.

In the next section, we further introduce IP as it relates to transdisciplinary co-design and other forms of responsible innovation practices. In section three, we discuss IP as a fluid *boundary-requiring-and-producing object* and the implications of this within agrifood worlds, and relate this to located response-ability. In section four, we further describe our case and introduce our methods for data collection. In sections five and six, we empirically describe potential and emerging IP-induced tensions within the MaaraTech Project: first noticing the specter of IP-induced tensions in interviews with project team members, and next through articulating the practices involved in protecting three potential IPs within the project. In section seven, we discuss how these empirical findings might be problematic when evaluated as project outcomes, but how they demonstrate useful exercises in located response-ability. We end with a set of suggestions for the MaaraTech Project, and reflections on the further development and integration of located response-ability in transdisciplinary co-design projects, particularly those producing AgTech.

## IP and transdisciplinary co-design

IP is often argued to be a necessary evil in research and development, particularly in the form of patents which provide comprehensive protection under legally-enforceable intellectual property rights. In the words of MBIE (2021)—the MaaraTech Project’s funding body—intellectual property rights:

give creators and innovators the exclusive right, for a limited time, to control what others may do with their creations and innovations. This exclusive right is based on the idea that intellectual property rights give people an opportunity to make a return on their investment in creativity or innovation. It also provides an incentive for creative or innovative activity that might not take place otherwise. The benefits of this additional creativity and innovation are considered to outweigh the costs imposed on society by intellectual property rights.

As the start of the statement illustrates, intellectual property rights exist to ensure that ideas are attributed to those who have developed them, and that the authors of those ideas have power over how the ideas are used and who benefits from that use. These property rights are exercised through exclusion and exchange. The “costs imposed on society” mentioned at the end of the statement are in most cases based on assumptions that any costs (monetary or otherwise) that come from protecting an invention through legally enforceable private property rights are balanced by the possible benefits arising from the public disclosure of inventions—a balance that some scholars question in part because it is an assumption that is difficult, if not impossible, to measure (Biagioli 2019; van den Belt 2013). Produced through a series of quite rigid institutional processes, IP can be viewed as the commodified version of a scientific output. Because the economic value of IP is easy to assess, it often becomes a primary mechanism through which the outputs of a technology development project are recognized and valued. This ease of quantification can often result in an increased focus on the economic value of IP, as opposed to the societal value of a research process, or the generation of ideas with less quantifiable or commercial worth. As a result, it is often difficult to notice the potential costs to society and end-users that can emerge when the capturing of IP is prioritized as a central researcher responsibility in technology design projects.

At the same time, there is discussion in the academic literature about how IP induces tensions within collaborative technology development processes. Many of these discussions focus on addressing tensions within university—industry collaborations (e.g., Eve-Levesque et al. 2013; Gretsch et al. 2020; Kneller et al. 2014; OECD 2011; Okamuro and Nishimura 2013; Tidd and Bessant 2021). While this literature mentions how IP can disrupt these collaborations, the use of IP as a measure of academic achievement or project success often goes unquestioned or is outright encouraged, particularly in AI and robotics where patent numbers have been increasing rapidly since 2013 (Chandra and Liaquat 2019; Marot et al. 2005; Van Roy et al. 2020). The possible negative effects of prioritizing the capturing of IP as a central researcher responsibility in collaborations with non-industry partners are often left out of these discussions, although there have been suggestions about the need to focus co-design efforts on something other than capturing IP—e.g., social justice or customer service—depending on the particular project and its goals (Marot et al. 2005). This trend to overlook the possible social consequences of prioritizing IP is most starkly apparent in the choice to put IP at the center of vaccine development in response to the COVID-19 pandemic, which provides insights into the way that current IP regimes center the needs of developers over the needs of wider society (Oxford Analytic 2021).

The concerns and desires of non-industry collaborators are centered within scholarship advocating for more responsible, collaborative, democratic, anti-colonial, anti-racist, reciprocal, participatory, culturally appropriate and societally beneficial research and development processes (e.g., Akama et al. 2019; Benjamin 2020; Bjerknes et al. 1987; Costanza-Chock 2020; Hales 1994; Kloppenburg 1991; Liboiron 2017, 2021; Suchman 2002; Taiuru 2020, 2022; von Schomberg 2011). This scholarship advocates for including Indigenous peoples, possible end-users, members of wider society and settings of use within technology development processes, including decisions about whether or not a technology should be made at all (see de Hoop et al. 2016). In this literature, IP is often noted as something that can disrupt responsiveness within more collaborative and participatory forms of technology production (Fraaije and Flipse 2020; Stilgoe et al. 2013). Responses to this restriction range from statements on the need to develop alternative IP regimes and management strategies that better align with the tenets of responsible innovation (or RI) and responsible research and innovation (or RRI) (Eastwood et al. 2019; Stilgoe et al. 2013), to discussions on how to expand IP options by turning toward models from open source and free software (Douglas and Stemerding 2013; Kyng 2010; Kloppenburg 2010; König et al. 2015; van den Belt 2013), or potentially producing metrics for responsibility to guide IP decision-making processes (König et al. 2015). Others recommend skillfully and creatively using dominant power hierarchies (e.g., gender hierarches which devalue feminine aesthetics) to dissuade university research offices from wanting to patent a technology in the first place (Liboiron 2017). These suggestions represent important institutional mechanisms and navigation strategies that may support more responsible IP processes.

## IP, boundaries, and located response-ability

Boundaries are often used to describe how IP emerges, relates and causes relational disruptions within social and material worlds. Describing IP as the “fencing off [of] ideas,” Boyle (2002) highlights how IP is able to create boundaries within our common intellectual resources and shape how ideas are shared in ways that reflect Western conceptualizations of property rights. Boyle (2002, p. 23) refers to the widespread protection of IP as the “second enclosure movement”—the first enclosure movement referring to the wide-scale privatization of common lands in England between the fifteenth and nineteenth centuries. For Strathern (1996), IP-induced boundaries between “inventor” and “context” emerge through a purification process where the creation of IP requires “cutting” away the social and material relations that participated in its emergence, but whose complexities do not fit within the confines of a patent application. As Carolan (2010) points out, while the boundaries created to protect IP and the property rights of its owners are designed to appear stable and immutable, they are often quite fluid in practice—particularly when dealing with lively, biological IP such as genetically modified organisms. He argues that this creates a situation where the rights afforded to the owners of IP might cut across (and disrupt) the rights afforded to land owners. Such a situation creates a need for “boundary work,” i.e., the discursive work (often in legal documents) that distinguishes between a potential, failed, or protected IP, as well as the property rights of IP owners and those who become their customers—whether intentionally or not (Carolan 2010).

To better understand how IP-induced boundaries affect social and material relations within a transdisciplinary co-design project, as well as how researchers participate in creating and navigating these boundaries, we build on these previous literatures by describing IP as a fluid *boundary-requiring-and-producing object*: an object that either requires or produces boundaries within social and material relations, depending on its stage of development. For example, potential IP requires specific boundaries (e.g., no public disclosure) in order to have a chance at becoming protected IP; protected IP creates boundaries between owners and users which are legally enforceable through intellectual property rights. In these ways, boundaries are not an *effect* but *inherent* to IP: they cannot simply be removed so that collaboration can proceed. We also refer to these boundaries as fluid due to their shifting nature and nebulousness. They tend to remain invisible and unarticulated in the everyday work of transdisciplinary co-design, often only becoming palpable when there is a boundary-crossing within a particular relational encounter—which might emerge as a tension within that encounter, or later when an encounter is reflected upon by an IP lawyer or patent owner. As technology development projects are often dealing with both potential and protected IP, it is important to be able to notice the boundaries required or produced by these different objects, how researchers are impelled to create or enforce these boundaries (sometimes contractually or legally required), and what can be done to prevent these boundaries from negatively impacting co-design processes.

Our description of IP as a *boundary-requiring-and-producing object* distinguishes it from discussions of “boundary objects” which “are plastic enough to adapt to local needs and constraints of several parties employing them, yet robust enough to maintain a common identity across sites” (Eastwood et al. 2012; Klerkx et al. 2012; Star and Griesemer 1989, p. 393). While a boundary object has so-called interpretive flexibility (Star and Griesemer 1989) and is able to move across boundaries to be interpreted differently by different groups of people in different settings, IP’s interpretation is not flexible. Hence, IP displays what has been dubbed “interpretive rigidity” (Klerkx et al. 2012, p. 39): the power to interpret IP is available to only a few—e.g., a judge or lawyer able to participate in official boundary work to decide on and define legal rights and protections (Carolan 2010). Thus, the fluidity of IP as a *boundary-requiring-and-producing object* does not refer to fluidity of interpretation. Instead, it refers to the shifting, nebulous nature of the boundaries IP incessantly requires or creates at different stages of development or protection, and in ways that can be difficult to notice or navigate without theoretical tools or specialist knowledge.

Open source and free software have been recommended by some scholars trying to seriously reckon with the boundary-producing nature of patents and other more closed forms of protected IP (Kloppenburg 2010; Kyng 2010). However, critically addressing IP-induced tensions involves acknowledging that these options are not silver bullet solutions, since using the structure of intellectual property rights to combat the ills caused by intellectual property rights is akin to, as Audre Lorde (1984, p. 113) so poignantly put it, using the master’s tools to try to dismantle the master’s house: while these tools “may allow us to temporarily beat [the master] at his own game, […] they will never enable us to bring about genuine change” (see Kloppenburg 2014). Re-designing data flows might similarly be promising for changing these extractive relations, but might also have similar constraints (Beckwith et al. 2019). Nevertheless, if public sector research is organized around proofs of concept and its associated IP, important tools for ensuring equity (e.g., in data handling) are too easily seen as a “use phase” matter for industry partners or open source developers (depending on the IP route taken) to decide later without public sector inputs. Whether the IP is left to open source or industry, leaving crucial matters to the “use phase” undermines the capacity of the public sector to reach non-commercial goals for scientific research.

An expansion of more responsible and democratic collaborative engagement with farmers in “upstream” technology design has also been recommended as ways to address the enormous socio-ethical and technological challenges facing the production and adoption of new AgTech (Bronson 2019, p. 1; Carolan 2010; Eastwood et al. 2012; Kloppenburg 1991; Lindblom et al. 2017; Lundström and Lindblom 2018). This includes engagements guided by the responsible innovation framework—attending to the dimensions of anticipation, inclusion, reflexivity and responsiveness in technology development processes (Bronson 2019; Eastwood et al. 2019; Rose and Chilvers 2018). These authors warn that collaboration is not a simple check-box (Lindblom et al. 2017) that can transfer all of the problems away from a bad “technical fix”—a top-down approach guided by industry—to a good “participation fix”—a more bottom-up approach (Black 2000, p. 496). As we argue in this paper, doing transdisciplinary co-design well will require noticing and navigating the boundary-requiring nature of potential IP within technology design processes—usually only noticeable through tensions emerging within the relations of technology production—and finding opportunities to address IP-induced tensions in ways that create a better balance between public and private interests (Eastwood et al. 2019).

Interested in offering practical solutions to bridging the divide between the “relations of production” and “relations of use” in technology design, Suchman (2002, p. 98) offers located accountability as an analytic tool and method to encourage researchers engaged in technology development to become more accountable to the sites where their technologies will be used. For Suchman, becoming more accountable to the relations of use will necessarily involve researchers crossing the boundary between technology production and use—i.e., engaging in more collaborative and participatory design processes. In transdisciplinary co-design projects, researchers are already crossing the boundary between sites of technology production and sites of technology use, though—as we have highlighted above—this is not the only boundary they are forced to contend with.

As boundaries around the relations of production expand with the introduction of transdisciplinary co-design, so do researcher responsibilities (e.g., to be responsive to the needs of research collaborators). However, a researcher’s abilities to be responsible will depend on their particular abilities to respond (response-abilities) within everyday research encounters and the institutional setting they operate within (Glerup et al. 2017; Liboiron 2017; Regan 2021). As researchers move from laboratories out into relations and sites of use, they will be forced to contend with these new responsibilities (and their transforming response-abilities), as well as any boundaries existing and emerging within these new relations. The inclusion of previously excluded actors introduces a number of new (or previously ignored) concerns, ethical considerations and power dynamics that also need to be contended with in practice (Borch and Throne-Holst 2021; Burch and Legun 2021; da Silva et al. 2019; de Bakker et al. 2014; Fritz and Meinhertz 2020; Nathan 2015; Ribeiro et al. 2018). As Regan (2021) has argued, practicing more responsible forms of research and innovation will require researchers build new competencies and undergo changes in mindset. However, as Glerup et al. (2017) has noted, researcher commitments to producing socially relevant and beneficial research outcomes mean little if they are encouraged by their institutional setting to prioritize patents and industrial partnerships in order to keep careers afloat and research institutions funded within the current political landscape of academic capitalism.

Our extension of located accountability to located response-ability is to provide a conceptual tool and practical method for researchers navigating new and shifting boundaries within widening relations of technology production. This is particularly important in fields such as robotics and human–computer interaction where “boundaries do not sit still” (Draude 2020). And even more important when *boundary-requiring-and-producing objects*, such as IP, are active participants within these relations. In this paper, we offer located response-ability as a conceptual tool and practice to support researchers engaged in transdisciplinary co-design to practice noticing and navigating the fluid *boundary-requiring-and-producing* nature of IP, and the ways it induces tensions in their everyday research encounters.

## The case

### Context

The MaaraTech Project is located in Aotearoa New Zealand. Māori are Indigenous people of Aotearoa New Zealand and their sovereignty, land rights and citizenship rights are legally recognized and protected under Te Tiriti o Waitangi (the Treaty of Waitangi)—a 1840 treaty which formalized the relationship between the British Crown and Māori and marked the establishment of New Zealand as a nation state (Hudson and Russell 2009; Solomon 2004). As a way to officially recognize their obligations under Te Tiriti o Waitangi, MBIE now requires all funding applications to adhere to its Vision Mātauranga Policy which necessitates the inclusion of Māori knowledge, scholars, communities, and interests within MBIE-funded projects (Burch et al. 2022; MBIE 2011, 2018; Muru-Lanning 2017).

When thinking about IP-induced tensions within technology development processes taking place in Aotearoa New Zealand and other non-Western, settler-colonial or post-colonial contexts, we must recognize that intellectual property rights are based on and reproduce Western conceptualizations of private property and ownership which are not universal (Garrity 1999). In the words of Māori scholar Aroha Mead (2002), cultural and intellectual property rights can represent “﻿the second wave of colonization ﻿because the principles that underpin western legal perceptions of particularly intellectual property are seen as a continuation of the ideologies of foreign conquest and domination.”

In recognizing a need to address these ongoing IP-induced tensions, official deliberations have finally been initiated to design new legal frameworks to protect taonga (treasures) and mātauranga Māori (“the body of knowledge originating from Māori ancestors, including the Māori worldview and perspectives, Māori creativity and cultural practices”) (MBIE 2019; Rauika Māngai 2020, p. 46). According to MBIE (2019), who plays a role in these latest negotiations, these new frameworks are expected to move beyond, though remain in alignment with, dominant Western IP regimes. This context makes the identification of IP processes and their current limitations particularly salient.

MaaraTech was funded through MBIE’s 2018 Endeavour Fund, making it a publicly funded project which must also secure co-funding from industry partners. The project’s goals and team were assembled in response to MBIE’s funding requirements of: demonstrating excellence in science and team make-up; producing impacts with clear implementation pathways; creating benefits for Aotearoa New Zealand; and complying with the Vision Mātauranga Policy. Transdisciplinary co-design was adopted as a method to achieve these funding goals and to produce industry-relevant, socially beneficial technologies.

Project actors include a core and fluctuating membership of over fifty engineers, computer scientists, agronomists, Māori scholars, social scientists, agricultural consultants, postgraduate students, interns, data labelers, administrators and IP lawyers located across eight universities, research centers and organizations. Research tasks are divided between the Technology Research Aim (technological development by engineers, computer scientists and others), the Community Technology Adoption Aim (social scientists responsible for studying the possible adoption of the technologies being developed) and the Māori Engagement Aim (Māori scholars responsible for studying the cultural aspects related to technology use and adoption). Central research collaborators include industry partners from the winegrape, apple, blueberry and robotics manufacturing industries, and growers from the winegrape, apple and blueberry industries. While agricultural workers are slowly being included within the project, they are being included through interviews and usability studies, and not the annual co-design workshops (described in more detail below) (see Burch and Legun 2021; Burch et al. 2022).

There are four main locations where our transdisciplinary collaborations take place: Industry Advisory Group meetings, co-design workshops, fieldwork visits and field days (described below). Some of our industry collaborators are members of the project’s Industry Advisory Group. IP agreements between the project’s lead University and industry partners, as well as non-disclosure agreements, make the quarterly Industry Advisory Group meetings a protected space where possible IP can be openly discussed. The project interacts with growers at annual co-design workshops and during regular fieldwork visits. The co-design workshops have porous boundaries to encourage any interested growers to join or invite their colleagues. The porosity of these boundaries means these meetings are not protected spaces where open discussions about possible IP can take place. Fieldwork visits take place throughout the year, which involve data collection (scanning plants and chatting with growers during group fieldwork trips for those working on the Technology Research Aim, and conducting interviews for those on the Community Technology Adoption and Māori Engagement Aims). Field days are opportunities for the project team to invite growers and industry partners to see technologies in action on local vineyards and orchards. The porous boundaries around fieldwork and field days means these are also spaces where discussions about potential IP are not able to take place.

### Methods and data

Data and inspiration for this paper comes from five major sources: (1) interviews with 36 project team members, 39 growers, and six industry representatives; (2) participation and observation at project meetings (quarterly Industry Advisory Group meetings with industry partners and co-funders, three annual industry-specific co-design meetings with growers, fortnightly technical meetings and monthly research management meetings); (3) participation and observation at annual team data-collection fieldwork trips and field days organized on orchards and vineyards; (4) information about the project and IP regulations stored on the project’s shared drive; and (5) clarifying conversations with inventors of potential IP within the project. Part six of this paper is essential to support the cultivation of located response-ability, as the people involved in the ethnographic descriptions were given an opportunity to contribute to or edit the observations made by the authors. Interviews, participation and observation all took place between 2019 and 2021, in the first 2.5 years of the five-year project.

Data was collected by the first (Burch) and third (Legun) authors who are both social scientists leading and contributing to the project’s Community Technology Adoption Aim. As embedded social scientists, they are both participants and observers in the relations of production that make up the project (Borch and Throne-Holst 2021; Suchman 2002). This positionality provided opportunities to gain an understanding of the particular IP-related aims and accountabilities (relevant for all team members) and IP-related developments within the project, as well as how others on the team understood and navigated IP-related issues in past projects and in real time. They listened to people’s thoughts and experiences, and observed practices of IP protection without judgment about what was *right* or *wrong* (Kemmis and McTaggart 2000), but to notice what was happening, how it was affecting the research collaboration, and the possible effects this might have on co-design, technology development and community technology adoption. The paper’s second (Nafus) and forth (Klerkx) authors—each dealing with IP-induced tensions in their own work as researchers within industry and university settings—contributed further support for critically reflecting on and challenging the observations made by the researchers embedded within the project.^2^

## The specter of IP-induced tensions

During interviews with project team members, IP consistently emerged as a point of tension that people working on the Technology Research Aim needed to contend with on a regular basis, both within MaaraTech and their other projects. In this section, we show how researchers working on the Technology Research Aim describe IP-induced tensions affecting their everyday work, and how these reflections point to the specter of further IP-induced tensions which, left unchecked, might affect the project and its outputs.

To begin, Elliot (a senior researcher working on the Technology Research Aim) described IP as a “stranglehold” affecting work within academia:

[IP] puts a real stranglehold on what we can do. You can continue to innovate technologically, it’s just that in an academic environment it simply doesn’t work. Well, it creates a lot of stress. […] You have to be particularly careful when you involve PhD students in these projects because they cannot operate under those constraints.

Through the lens of located response-ability, Elliot’s description highlights how the boundaries required to protect IP have negative effects on his desire to include PhD students within his projects. Here we can see a tension between funding guidelines and research practices: postgraduate students are required within MBIE-funded projects to support the capability-building of emerging scholars and practitioners, and IP can disrupt the intellectual and professional development of these students by restricting their ability to engage in open scientific debate and to share their findings with colleagues in their scientific disciplines. Gordon (a PhD student working on the Technology Research Aim) described some of the “disruptive” effects IP has had on his postgraduate colleagues:

[My colleagues] are looking at getting some patents or some sort of design registration or something on their designs and that is disruptive to presenting and sharing and essentially getting further input from other people on the designs.

Hugo (a senior researcher working on the Technology Research Aim) also discussed the “disruption” IP brings to scientific practices and scientific culture more generally:

…science needs discussion to be, yes, flourishing. It needs to have creativity and all this comes only from discussing it. And, if you just disclude anything, you don’t talk with your colleagues anymore about what you did yesterday because you are afraid that he may learn something that he can publish before you do it. Or he patents it. It’s a disruption in this culture that is very, very difficult.

Located response-ability allows us to notice IP’s *boundary-requiring-and-producing* nature and how IP is a disruptive actor within research processes. More specifically, it provides insights into how the boundaries required and produced by IP affect how scientists relate with each other in everyday encounters: their relations and response-abilities within these relations are guided by the need to protect IP. This creates a situation where researchers may find it difficult, or contractually prohibitive, to collaboratively come up with creative solutions through open and transparent dialogue with people beyond one’s project team members.

Others discussed the importance of and “constraints” that come with the necessity to capture and protect IP within industry co-funded projects—or any project wanting to commercialize technologies. In particular, Stan (a senior researcher working on the Technology Research Aim) described the specter of losing industry partners if the project fails to put the required boundaries in place to protect potential IP that may be of interest to industry partners:

We may not achieve the overall goal if [team members] let all the IP leak out […], because companies need to have the IP contained so they can get further investment, which is one of the parts of transferring the technology if you like. And so you can lose your partners and lose your pipeline for translating it into something useful if you do that. So there’s constraints about that.

Ron (a senior researcher working on the Technology Research Aim) also highlighted the importance of protecting IP for industry partners, though points to an additional IP-related tension: the specter of IP negatively affecting relations with growers, the robustness of technological outputs and possible technology adoption. He described this tension as a “delicate line” that needed to be navigated when trying to protect IP at the same time as researchers remain responsive to the needs of growers and other collaborators:

This is quite a delicate topic because of all the IP issues that we have been dealing with, for example. So when we want to do all our testing at the farm, and realize we can’t really show this to the growers. But then if we don’t show it to the growers then we haven’t actually tested it on the farm. So, what is the delicate line in saying, ‘ok we can use your farm but do not come into our boundary.’ That doesn’t make sense, because if they do not see the thing and how it works, or doesn’t work, then how do you know whether that’s what they’re actually expecting? But then the IP, I understand, is important, because there’s a company that’s interested in this and they’re wanting to bring it along further. They have to make sure it’s IP protected, and those are really delicate issues.

Viewed through the lens of located response-ability, these descriptions provide initial insights into how the *boundary-requiring-and-producing* nature of IP can affect the everyday work of researchers engaged in technology design projects, and how IP becomes a central actor shaping design processes as it impels researchers to create and navigate the boundaries it requires and produces. The reflections also highlight how team members’ abilities to respond (response-abilities) within the relations of technology production and transdisciplinary co-design are ever-affected by the presence of potential and protected IP in their everyday work (and the boundaries these different forms of IP require and produce). Technologies need testing on farms, researchers need open interactions with farmers, and students need open-ended discussions with other scholars. However, IP enters these relations and impels researchers to transform their desired responses to fit its needs. These findings link to other scholarship discussing the tensions that can arise when researcher responsibilities to contrasting economic and social aims create tensions within everyday research encounters (Glerup et al. 2017; de Saille 2015). They also align with scholarship describing the mundane, structural enablers and barriers which affect researchers’ abilities to fully enact forms of responsible innovation (Liboiron 2017; McCampbell et al. 2021b). Exactly what the navigation of IP-induced tensions looks like in practice, and the located response-abilities available to potential IP developers within the MaaraTech Project, will be explored in the next section.

## IP in Practice

### FastAnnotation

FastAnnotation is a software tool developed by junior project researchers to speed-up annotation processes through supporting annotators with detecting objects of interest (e.g., apple fruitlets) within visual data collected from farms. The software was first presented within the protected space of the project’s Industry Advisory Group meeting in early 2020. In presenting the possible IP, its inventors requested for the software to be protected through an open source license. The rationale behind this request was due to the particular way its inventors, and the project, conceptualized the materiality of the potential IP (a software) and its purpose (designed to speed-up annotation processes within the project). This desire to get on with the technology design process (i.e., to create technologies that could detect objects of interest) meant that quickly open-sourcing the software would have supported project accountabilities to:

- *project funders: *ensuring outputs are on time;
- *advancing science:* allowing technology design processes to continue;
- *capabilities building and advancing careers:* junior scholars could add the open source software to their CVs;
- *producing and protecting IP:* open source is a form of IP;
- *commercial relevance: *software was not considered to be of commercial interest, but to support the team to continue working toward their proof of concept for potentially commercializable technologies; and
- *collaboration: *supporting the internal collaboration among researchers working on the project’s Technology Research Aim.

In the end, the software was open-sourced under the General Public License version 3.0 and shared on Github where other project team members and members of the wider scientific community could access and use it (UoA-CARES 2020). The Copyleft license allowed the team to share complete source code as well as modifications. This meant that copyright was reserved, but all patent rights were relinquished.

Thinking through located response-ability, the boundary needed to protect the potential IP during the deliberation process did not disrupt the wider transdisciplinary collaboration or interfere with other project aims or accountabilities as the main concern was about ensuring team members could use the annotation tool for technology development. Also, as software is notoriously difficult to patent, the decision to open-source was straightforward and easy. However, as we will highlight in section seven, by not locating FastAnnotation withing the wider relations of the project’s transdisciplinary co-design processes, the team inadvertently overlooked other possible uses for this potential IP, which could have benefitted both industry partners and growers.

### The Barracuda

The Barracuda is a pair of secateurs designed by a junior engineer working on the project’s Technology Research Aim. The hardware was designed with a “barracuda” blade to support the pruning of winegrape vines without cutting through wires. The idea emerged from discussions with growers who had described unintentional wire cutting as a potential issue that could arise in automating this task. It was first presented as a potential IP in the protected space of the Industry Advisory Group meeting in early 2020. At the time, it seemed to support the project’s accountabilities to:

- *project funders:* a useful deliverable;
- *advancing science: *producing a novel solution;
- *capabilities building:* it was produced by a junior engineer;
- *producing/protecting IP:* it represented a possible patent; and
- *commercial relevance: *industry partners in the room were excited by the invention.

In the end, the hardware’s design was protected by copyright and its use protected by freedom to operate (the ability to continue using the design without worry that others will see it as an infringement on their intellectual property rights). The Barracuda was ultimately found to not be patentable because it did not meet the requirement of being new/novel, clever or unusual: there were other patents presenting a similar idea (even though the use and design of the Barracuda were distinct). However, in discussions about the IP decision, it was mentioned that because iterated versions of the hardware were shared at co-design meetings (where attendees had not all signed non-disclosure agreements), this would have disrupted the patenting process had the hardware fit the patenting criteria.

Thinking through the lens of located response-ability, in this case the engineer’s responsibility to protect potential IP was in tension with his responsibility to the transdisciplinary collaboration (responsiveness to growers) and, thus, toward commercial relevance (without collaboration with the growers, it would be impossible to create the most relevant technology for industry to commercialize). That is, the engineer continued to collaborate and innovate responsively with growers, but in doing so did not maintain the boundaries necessary to protect the potential IP that emerged. While in the end the engineer’s responsiveness within the project’s transdisciplinary co-design process did not have an effect on the patent itself, the Barracuda’s journey points to potential issues moving forward as more potential IP begins emerging within the project. As we will discuss further in section seven, using the framework of located response-ability to situate IP as a fluid *boundary-requiring-and-producing* actor within the project’s wider collaborative relations could support researchers in better navigating IP’s shifting boundaries in their everyday work. This awareness will be necessary to reach the project’s aim of community technology adoption: to create technologies that are relevant to industry and growers and which they want to adopt.

### Four Pedals

Four Pedals is another hardware, in this case designed by a postgraduate student working on the project’s Technology Research Aim. The hardware was designed to prune apple fruitlets. Like the other examples, Four Pedals first emerged within the protected space of the Industry Advisory Group meeting in early 2020. In this case, the hardware received a provisional patent. Around the time its provisional patent was expected to expire (a year after it was first filed), the project had—in alignment with project contracts—asked industry co-funders if anyone wanted to patent it. This was because the project itself requires industry partners to pay for the patenting process.

A confirmation email was sent to the research management team and industry partners to inform everyone that there was no industry interest in patenting Four Pedals, and that a patent would not be sought. One of the project’s industry partners had concerns that this indicated a boundary between researchers and industry that was hindering the capacity to produce industry-relevant technologies. Earlier, he had advocated for more responsiveness between technology developers and industry, recommending the creation of Buzz Groups—regular check-ins between industry partners and researchers working on the Technology Research Aim, taking place outside of the official Industry Advisory Group meetings which had been disrupted throughout 2020 due to the COVID-19 pandemic. The industry partner had suggested Buzz Groups as a method to ensure the technologies being produced remained relevant to industry’s needs, which might shift depending on internal or external circumstances. In hearing the news about a lack of industry interest in Four Pedals, he again asked why the Buzz Groups were not yet happening.

Discussions among the team highlighted that there are many reasons why industry might not be interested in a single end-effector at this point in the project (it has not yet been attached to a robot or turned into a hand-held device to support agricultural workers). However, in engaging in discussions and thinking with located response-ability, some of the other reasons why Buzz Groups had not yet emerged became clearer: potential IP requires clear boundaries to protect it, and the casual, potentially-porous nature of Buzz Groups—where junior scholars would be meeting with members of industry, and where there may be no minutes taken or no clear indication of who would be responsible for maintaining the boundaries required to protect potential IP—complicated their emergence.

As with the Barracuda, the journey of Four Pedals highlighted how researchers’ responsibilities to protect potential IP were in tension with their responsibilities to remain responsive to the needs of industry and, thus, toward the development of commercially relevant technologies. However, thinking through located response-ability, while the establishment of Buzz Groups might have supported responsiveness between researchers and industry partners, the establishment of additional boundaries would have been required to ensure the meetings were protected spaces where potential IP could be discussed without threatening its potential patentability.

## Discussion

While the abovementioned empirical findings may seem problematic when evaluated as project outcomes in a quantifiable sense (i.e., they do not represent patented IP that can be translated directly into commercial value), they do demonstrate useful exercises in located response-ability: they provide opportunities to notice IP’s fluid *boundary-requiring-and-producing* nature in everyday research practices and how these boundaries were, or could have been, navigated. In examining these experiencess and cases in this way, we can better consider the role IP plays in transdisciplinary co-design in terms of establishing or breaking particular relationships of responsibility, and in ways that fluidly shift depending on the particular relations one might be relating within in a particular place, on a particular day or at a particular point in time. This provides insights into how we can support more responsible AgTech development by more consciously and assertively establishing IP processes that produce the relations of responsibility we advocate and strive for in transdisciplinary co-design projects.

The IP-related experiences and practices shared in sections five and six illustrate that an inability to skillfully navigate the boundaries required and produced by IP could lead to a number of consequences in technology development and transdisciplinary co-design, such as: impeding the professional development of postgraduates; decreasing scientific creativity; disrupting responsiveness with industry partners; disrupting responsiveness with growers; and potentially disrupting the technological robustness and adoptability (societal relevance, benefit and acceptability) of the technologies being developed. As there is no escaping IP’s boundaries within technology design processes—at least within conditions of academic capitalism—these observations highlight that researchers hoping to practice more responsible innovation require conceptual tools and practices to support them in noticing and navigating these fluidly-shifting boundaries in everyday practice, particularly when working within the dynamic relations of a transdisciplinary collaboration.

Within the MaaraTech Project, team members’ responsibilities to protect IP are currently communicated through employment contracts, as well as presentations and guidelines given by the lead university’s research office. These presentations and guidelines describe different types of IP and the team’s collective contractual obligations to protect it for industry partners, with a focus on IP creation and protection (e.g., the need to disclose potential IP to the research office whenever it emerges; to not publicly disclose potential IP before it is legally protected). Maintaining relations with industry partners who need IP to be protected for future commercialization purposes are central to the presentations and guidelines, with no additional discussions about the possible effects IP protection (i.e., boundary creation) might have on everyday scientific practices, co-design processes or the wider societal benefits of technologies. Prioritizing researcher responsibilities to protect potential IP may create a shared goal which focuses research collaborations (Marot et al. 2005). It may also support transdisciplinary co-design projects that only extend to industry partners (i.e., where contracts and non-disclosure agreements create protective spaces for open IP-related discussions) (e.g., Eve-Levesque et al. 2013; Gretsch et al. 2020; Kneller et al. 2014; Okamuro and Nishimura 2013). However, transdisciplinary co-design poses a number of new challenges to researchers who will be forced to create and navigate IP-induced boundaries depending on whether a particular interaction is protected for IP-related discussions or not. This suggests the need to expand IP protection guidelines to better support researchers in noticing and navigating these fluidly-shifting boundaries within their everyday work, and strategically negotiating these boundaries to make meaningful and equitable inclusion possible. While we agree with responsible innovation scholars that sweeping, uncritical calls for inclusion can be problematic (see van Mierlo et al. 2020; McCampbell et al. 2021a), our findings indicate a need to be aware of where interpretive rigidity of IP and the institutional context that maintains it can become (perhaps unintentionally) problematic so that even transdisciplinary co-design becomes exclusionary and unresponsive to the needs of research collaborators (see also Liboiron 2017; McCampbell et al 2021b).

In this paper we illustrate how located response-ability can support the process of locating IP and IP-related practices within the relations that make up a transdisciplinary co-design project, with each IP decision and practice providing an opportunity to further develop skills in noticing and navigating IP’s fluidly-shifting boundaries. This exercise also illuminates the ways that IP promotes particular responses between actors (human and more-than-human), while potentially foreclosing others. Being conscious of how IP affects response-abilities within a transdisciplinary co-design project can support more responsible innovation by highlighting the possible consequences of these choices on responsiveness with research collaborators and wider societal actors. If transdisciplinary co-design strives to be responsive to the needs of societal and industry actors, it is important to take opportunities to support the possibilities of that responsiveness. As we describe below, IP provides an everyday opportunity to interrogate and strengthen approaches to responsiveness within technology design projects, particularly those using transdisciplinary co-design to increase the societal benefit and relevance of technologies.

In the examples of the Barracuda and Four Pedals, we can see how researchers’ responsibilities to protect IP and their responsibilities to be responsive to the needs of research collaborators (growers and industry partners) can be put in tension with each other. What seems to be at issue here is the implicit, unstated default prioritization of IP creation and protection at the possible expense of other stated project aims of community technology adoption and Māori engagement. This seems to be a prioritization that emerges in practice, and not as a matter of stated project goals or deliverables. In any given moment, researchers appear more able (and pressured) to respond to the needs of potential IP than they are to respond to the needs of other participants within the transdisciplinary co-design process. Thus, the prioritization of IP seems to emerge as a default not because of any deliberate choice made by project team members, but because of a broader context of academic capitalism and dominant scientific culture where IP is a default (more-than-human) actor in all scientific processes, whether researchers want to engage with it or not (e.g., Carolan 2010; Glerup et al. 2017; Hackett 2014; Kloppenburg 2014; Liboiron 2017; de Saille 2015).

Through noticing these defaults, and who/what researchers are capable of responding to in any particular setting, we can begin to notice which other researcher responsibilities and stated project aims and accountabilities enjoy less attention (and, thus, less response) in practice. We can then also ask what alternative responses *could have been possible* within the three potential IPs we observed. In Four Pedals, an industry partner himself suggested a processual remedy that could have led to a different technical direction. In the Barracuda example, if the cultural default was centered on responsiveness with growers, then the discussion might not have been about whether sharing the hardware at a co-design workshop constituted a possible invalidation of IP, but more about how many growers need to be asked to sign non-disclosure agreements, or be listed on the patent disclosure as co-inventors, and how to handle the consequences of doing so. Such collaborative IP management could potentially play a role in creating more equitable relations between technology and data owners and technology users and data producers, transforming current trends in which the adoption of AgTech leads to widening power asymmetries within agrifood worlds (e.g., Bronson 2019; Bronson and Knezevic 2019; Bronson and Sengers 2022; Carbonell 2016), and perhaps even opening space for emancipatory, anti-colonial or commons-based approaches that critically address questions of food and data sovereignty (e.g., Beckwith et al. 2019; Fraser 2019, 2020; Kloppenburg 2014; Taiuru 2020, 2022).

The FastAnnotation tool seemed to be the least problematic example, yet in many ways shows most clearly the societal and economic costs of prioritizing IP as a central researcher responsibility in technology development. In FastAnnotation, developers conceived of the software as relevant only to the internal research team and other AI developers like themselves (hence, the GPL license). If the developers had prioritized responsiveness and included growers as a source of expertise of how to appropriately label plant parts, this might have changed their commercial position, and made the software of higher value to industry partners and AgTech firms—who might have seen value in incorporating such tooling in the systems they ultimately build, and the value of labels from growers themselves. It is possible that the inclusion of growers at the point the IP-decision was made might have opened-up useful conversations about how growers do and do not want to be able to *teach* the robots on their farms how to work. Thus, prioritizing responsiveness to growers could have led to unanticipated social and economic benefits.

Typically, AI systems do not work well without in-situ retraining, which often requires robotics vendors, their suppliers, or even their customers, to label new data (Thomas 2019). Therefore, even leaving aside questions of societal benefits, there is reason to believe there is commercial value in an annotation tool optimized for the needs of agricultural applications. Thinking through located response-ability, we can see that the potential to commercialize FastAnnotation was not considered since IP was being imagined in a particular way—as a patentable computer vision-enabled robotic system—and researcher responses were organized around that imagination. Such an imagination allows annotation to be considered an activity that happens on the way to the “use phase,” and therefore growers, who were considered “users,” did not appear relevant to that process. The fact that it was software, usually protected by copyright rather than patent law, similarly made FastAnnotation less relevant as an IP output, but more relevant to other technology developers within the project who write and use code in equal measure. In these ways, researchers’ accountabilities to advance science and engage in internal collaboration with project team members, paired with their knowledge on the difficulty of patenting software tools, organized their responses in ways that reduced their abilities to recognize other commercial possibilities or societal benefits for the software not listed within the project’s pre-determined research aims and milestones. Here, beneficiaries were assumed to be other developers, which in turn created a boundary between what was imaginable as a production-phase tool and what was imaginable as a software feature for the use phase. In this case, the unexamined boundary between who is a producer and who is a user could have reduced the overall value of the potential IP.

While in this example Māori growers were not included in the development discussion—no growers were—the fact that a data-gathering robot could end up on Māori-owned vineyard or orchard adds additional questions about data sovereignty (Walter et al. 2021) and ethical considerations for aligning collaborations with Te Tiriti o Waitangi (Taiuru 2020, 2022). A data annotation tool compatible with meeting obligations to Indigenous data sovereignty might have significantly different features, perhaps more elaborate tooling for data provenance, categories of what is labelled and unlabeled, among other features. However, these aspects would only be discoverable if responsiveness with research collaborators and wider societal actors is actively prioritized and practiced within technology development projects.

Located response-ability insists on the acknowledgement of who/what researchers are responding to, and who/what is being excluded or neglected in any given moment—even as researchers maintain relations with those they are not currently responding to. This acknowledgement can help researchers to reflect on whether it is truly the case that they lack the *ability* to respond to those they are currently excluding, or if it is their sheer attention to other responsibilities—and the cultural “default” settings that call attention to some matters over others—that is inhibiting their abilities to respond. It also makes located response-ability a tool that can support researchers in better conceptualizing their competing responsibilities, and to practically navigate their possible responses, and boundaries to those responses, within their everyday work. Thus, engaging with located response-ability provides an everyday opportunity for researchers to transform how they might imagine themselves as practitioners of responsible innovation (Regan 2021). It also provides opportunities to articulate, navigate and intervene in the various boundaries to practicing more responsible forms of innovation (e.g., institutional pressure to prioritize IP at the expense of research relationships), particularly within the context of academic capitalism (Glerup et al. 2017; Liboiron 2017).

These examples show that the societal costs paid to maintain an IP-dominant system—paid by beneficial technologies going unpursued, or by researchers pursuing technical directions out of direct-relationship with possible end-users or wider societal actors—is likely underestimated, particularly in the field of AgTech where end-users such as farmers and agricultural workers are often not considered or left out of technology design processes (Burch and Legun 2021; Rotz et al. 2019a, b). More broadly, these examples suggest that the solution is not merely about including the relevant actors “upstream,” or earlier in technology development processes, because IP incessantly *requires* and *produces* boundaries throughout these processes. Rigidly adding quarterly check-ins or milestones will therefore not be enough to support researchers as they try to navigate the consequences of these fluidly-shifting boundaries within dynamic collaborative relations. The answers must come from more consciously and assertively establishing IP processes that prioritize responsiveness in innovation. Until that happens, researchers committed to responsible forms of innovation must find ways to remain responsive to research collaborators as they navigate IP’s fluid *boundary-requiring-and-producing* nature. Located response-ability can support researchers to notice IP-induced boundaries and to more skillfully navigate these boundaries in the everyday work of technology design.

## Reflections and conclusion

In this paper, we have shown how IP is a fluid *boundary-requiring-and-producing object* that creates social distinctions and tensions within a transdisciplinary co-design project developing new AgTech. Our analysis empirically examined IP-induced tensions in interviews with project team members and through articulating the journeys of three potential IPs in practice. Through the lens of located response-ability, we have illustrated how unchecked IP-induced boundaries can potentially disrupt the collaborative research relations required to produce industry-relevant, socially beneficial technologies. In the examples, researcher responsibilities to protect potential IP created a situation where it was difficult to remain responsive to the needs and experiences of growers and industry partners collaborating with the project. This responsibility to capture and protect IP also narrowed opportunities to creatively and collaboratively imagine additional values, beneficiaries and uses of potential IPs emerging within the project.

While turning directly to possible solutions such as open source and free software may seem intriguing, we have shown that, while helpful, these tools do not fully solve the underlying social problems that emerge when IP enters transdisciplinary co-design projects. This is because these solutions maintain a focus on IP transferability (whether as closed or open IP) rather than finding ways to remain more responsive to research collaborators and wider societal actors while protecting potential IP. Thus, training researchers engaged in transdisciplinary co-design in how to more skillfully notice and navigate the fluidly-shifting boundaries required and produced by IP is potentially more powerful than any licensing toolkit or contractual language. This is because, regardless of how much one might want to avoid it, navigating IP’s boundaries is currently an unavoidable part of engaging in the everyday work of technology design in the context of academic capitalism. Transdisciplinary co-design—and the dynamic social relations it introduces—further complicates how and when these boundaries emerge, as well as their potential impacts on the technologies being developed.

Skills in noticing and navigating boundaries within collaborative processes are becoming more essential for researchers hoping to get technology design projects funded, since many prominent funding institutions are increasingly advocating for the capturing of IP *and* the production of societally beneficial and culturally relevant technologies in their funding calls (e.g., Borch and Throne-Holst 2021; MBIE 2018; Von Schomberg and Hankins 2019). Our analysis highlights that the boundaries required and produced by IP create a particular context which necessitates that researchers become responsible for noticing and navigating these boundaries in their everyday work. This is particularly the case for projects aiming to develop societally beneficial and culturally relevant technologies through methods such as transdisciplinary co-design, or those working with research collaborators who may not agree with Western conceptualizations of private property. Located response-ability is a conceptual tool and practice that can support researchers to better locate themselves and IP within the relations of technology production, allowing them to notice and navigate IP-induced boundaries without causing irreparable harm to collaborative research relations and, thus, the possible benefits and relevance of technological outputs. This may be one way to ensure that the societal benefits of new agricultural technologies can be generated, and positive science–society relationships prioritized and preserved as researchers try to practice responsible forms of innovation within the context of academic capitalism (Glerup et al. 2017).

As the adoption of new AgTech can lead to the widening of power asymmetries between AgTech, agribusiness and BigTech firms on one end and farmers and agricultural workers on the other, responsible innovation approaches are being advocated and tested (e.g., Bronson 2019). However, as scholarship in the field of responsible innovation has already indicated, responsibility is ultimately an ongoing process of everyday experimentation, where grasping what is truly at stake comes from trying to enact responsible innovation in practice (Eastwood et al. 2019; Fielke et al. 2021; Fleming et al. 2021; Lioutas and Charatsari 2021). Designing new AgTech through methods such as transdisciplinary co-design is a notable first step in attempting to create more equitable outcomes in AgTech. However, adding more actors to design processes will only lead to more responsible innovation if researchers have the conceptual tools and practical methods to notice and navigate their competing responsibilities, and any boundaries to their desired responses, within their everyday work. Alongside methods such as unravelling socio-cyber-physical systems (Rijswijk et al. 2021) and navigating one’s compromised agency (Liboiron 2017), located response-ability can support researchers in developing these skills, allowing them to better navigate IP’s boundaries in ways that transform these trends in inequity.

While we do not claim to solve the problems caused by IP-induced tensions, our observations lead us to suggest that there could be a way of integrating a located response-ability-informed practice for noticing IP-induced boundaries and making explicit decisions on how to handle them for each potential IP that emerges within a transdisciplinary co-design project. That is, transdisciplinary co-design projects could adopt a rubric for acknowledging issues and identifying alternatives beyond the default dominance of IP. This could begin with new forms of expectation setting: in the same way that universities formally define researchers’ responsibilities to protect IP in presentations and workshops, they could set expectations about researchers’ responsibilities to research collaborations and wider societal relations, and provide guidance and support for navigating IP-induced tensions in practice.

**Fig. 1:**
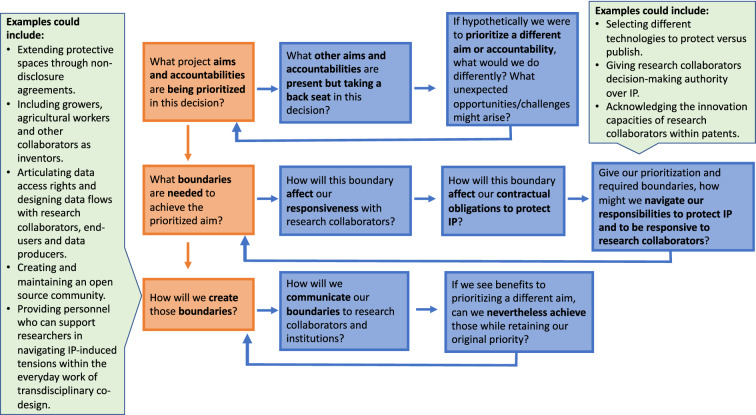
Example exercise in located response-ability for responsibly navigating IP-induced tensions

When it comes to key decisions (e.g., what to patent, or what to share and not share with research collaborators), we suggest that transdisciplinary co-design teams could benefit from engaging in open deliberations on the questions listed in Fig. 1 as an explicit exercise in located response-ability.

A reader might ask why these questions force choices instead of assuming that projects can meet their IP goals and societal commitments at the same time. This is not because we believe that there is an inherent conflict of interest. Instead, it is to highlight how unexamined assumptions that all project aims are equally prioritized is not necessarily useful, and potentially even threatens project success, as the existence of a problem can go unacknowledged or tensions simply endured for lack of awareness of possible alternative responses. Therefore, we need conceptual tools and practices such as located response-ability that help us to destabilize that assumption within the contexts where actual decisions are made and IP-induced boundaries are actively negotiated in practice. We also ask the hypothetical counterfactual question because, as we saw in the FastAnnotation example, reasoning through the consequences could identify unanticipated areas of innovation that are made invisible by the default priority. These might nevertheless have value in terms of that priority.

## Footnotes

1 Aotearoa is a te reo Māori name for New Zealand.

2 Although the second author (Nafus) works in an industrial context, her employer was not among the industry partners working directly on the project. Nafus’s contribution was strictly as a collaborating analyst.
